# Ground truth based comparison of saliency maps algorithms

**DOI:** 10.1038/s41598-023-42946-w

**Published:** 2023-10-06

**Authors:** Karolina Szczepankiewicz, Adam Popowicz, Kamil Charkiewicz, Katarzyna Nałęcz-Charkiewicz, Michał Szczepankiewicz, Sławomir Lasota, Paweł Zawistowski, Krystian Radlak

**Affiliations:** 1 Independent Researcher, Warsaw, Poland; 2 Department of Electronics, Electrical Engineering and Microelectronics, Silesian University of Technology, Akademicka 16, Gliwice, Poland; 3 Institute of Computer Science, Warsaw University of Technology, Pl. Politechniki 1, Warsaw, Poland; 4 NVIDIA, Warsaw, Poland

**Keywords:** Computational science, Computer science, Information technology, Scientific data, Software

## Abstract

Deep neural networks (DNNs) have achieved outstanding results in domains such as image processing, computer vision, natural language processing and bioinformatics. In recent years, many methods have been proposed that can provide a visual explanation of decision made by such classifiers. Saliency maps are probably the most popular. However, it is still unclear how to properly interpret saliency maps for a given image and which techniques perform most accurately. This paper presents a methodology to practically evaluate the real effectiveness of saliency map generation methods. We used three state-of-the-art network architectures along with specially prepared benchmark datasets, and we proposed a novel metric to provide a quantitative comparison of the methods. The comparison identified the most reliable techniques and the solutions which usually failed in our tests.

## Introduction

Convolutional neural networks (CNNs) achieve outstanding results solving problems in domains such as image recognition, computer vision and bioinformatics. However, deployment of a black-box model such as a CNN introduces uncertainty about the generalizability of the model and about which features were important for the model’s decisions. Even when the output of a classifier is correct, its decision might be based on the identification of the wrong set of features such as artifacts in the background of an image. This may happen when systematic bias is introduced in training data or when there are spurious correlations in these data^1^ that remain undetected during the development process. One of the most famous experiments illustrating an insufficient learning base was presented in^2^. Wolves had been systematically photographed against a snowy background, and a husky photographed in the same environment was misidentified as a wolf because the AI solution concentrated on the background while ignoring the intended subjects of the images.

Current state-of-the-art analyses and methods do not comprehensively address machine learning interpretability. They rather focus on a narrow subset of issues and, as a result, only limited guidance can be extracted. One line of work focuses on the taxonomy definition and emphasizes interpretability mechanisms. A broad overview of black-box-like algorithms has been presented in^1,3^. The authors outlined the deficiencies which should be addressed to ensure that the algorithms perform predictably. Some evaluative aspects have already been addressed in^4–6^, but that work does not exhaustively cover the interpretability of a DNN model, and applicability to safety-critical systems still remains an open question.

An attempt to introduce model interpretability and to use it to optimize performance was made by^7^. The approach was successful in reconstructing and visualizing features of the input image that had been identified by the intermediate layers of a network. The technique allowed for the improvement of CNN performance: it increased the network’s resilience against variations in image background and improved model’s focus on the local object structure.

The authors of^8^ presented two solutions that visualize both features and activations at each internal layer of the CNN model. The first solution was based on the fact that the correlation between neural activation patterns and training inputs can increase understanding of the model’s behavior and can improve the development process through the application of transfer learning techniques. The second solution was based on several new regularization methods. It proved useful in detecting weak spots in the coverage of the training set such as occurs when a network identifies a jaguar only by the spots on the fur, completely ignoring all other features.

Saliency maps are probably the most popular technique for providing visual explanations of the decisions of CNNs. As presented in^9^, the visualizations provided by this approach help explain the failure of CNNs, to identify biases present in the datasets, and to prepare models that are robust against adversarial attacks. They therefore offer an improved development process and greater generalization of trained models.

One important step toward techniques for comparing saliency maps was presented in^10^. The authors performed two types of randomization tests. The first focused on the randomization of a model. The second randomized labels in a training dataset to check the performance of saliency map algorithms on a correctly labeled test dataset in the context of a search of outliers. The saliency maps in presented experiments were compared using Spearman rank correlation with absolute value (absolute value), Spearman rank correlation without absolute value (diverging), the structural similarity index (SSIM), and the Pearson correlation of the histogram of gradients (HOGs). The authors found that some of the methods were independent of the model and data and were thus reliable. Others failed due to strong correlations between the generated saliency maps and the edges in the image data. Although such a methodology shows which saliency maps are strongly or weakly correlated with network training processes, it is still unknown whether the silency map highlights the correct areas of interest. Therefore, even with the methods indicated as reliable (i.e. the ones showing the weak correlation), the human operator can not assess which objects in the image made the largest impact on the final decision of a network.

In this paper, we analyze and compare the efficiency of saliency map based techniques that provide visual explanations for CNN decisions. The proposed experiments are performed in an integrated environment using several state-of-the-art network architectures and specially prepared and tagged datasets. In comparison to the work^10^, we establish a controlled environment in which the efficiency of the saliency map algorithms can be objectively evaluated and quantified using a novel metric. We also identify techniques that allow for the detection of systematic failures in image datasets or in the process of CNN training. Finally, we indicate which group of methods should be employed to reliably explain the relations between a CNN’s inputs and outputs.

## Methodology

For the proper evaluation of the efficiency of saliency maps algorithms, the use of any comparison method or metric needs to be justified. In the literature, several methods of saliency maps evaluation have been proposed. Saliency maps have been evaluated visually^9,11–14^, by a comparison of the ground truth images to the automatically, semi-automatically, or manually created masks^15^, or by the use of numeric methods^16^. In the work^16^, the authors applied the Remove and Retrain (ROAR) and Keep and Retrain (KAR) techniques^17^. However, such approaches require image segmentation with its exhaustive modifications and accompanying model retraining. This can be impractical for large datasets. Some of the methods dedicated to the evaluation of DNNs can be found in^18^, in which the authors present a library called *iNNvestigation* that they developed for testing networks in Python 2.0 or 3.0 using Keras.

**Figure 1:**
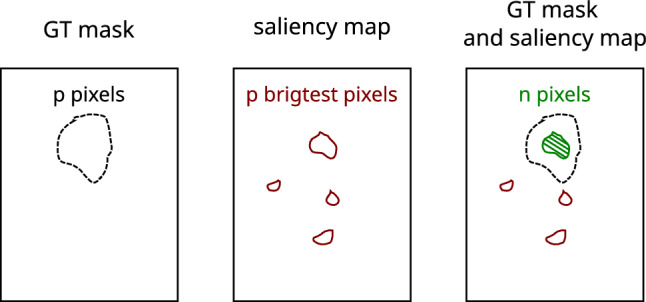
The idea of proposed new evaluation measure $m_{gt}$.

To evaluate a model’s efficacy at finding fragments relevant for classification via saliency maps, we applied a technique that is much simpler than ROAR or KAR. With this technique, binary ground truth (GT) masks are assigned manually or semi-automatically to each image in a diverse, specially prepared image database. The identification of the target region is possible thanks to the recognition of the objects responsible for an image being assigned to a given class. If we assume that a GT mask has *p* binary ones, in a saliency map generated for a given image, we select also the *p* brightest (i.e. most significant) pixels to create a saliency map mask. We then calculate how many of the binary ones align between the two masks. The proposed indicator compares the coverage of the most relevant pixels in the saliency map with the indicated pixels in the manually prepared GT masks. A graphical explanation of this new metric is presented in Fig. 1. The assumed measure is $m_{GT}=n/p$, where *n* is the number of pixels in the saliency map mask falling in the positive regions of a GT mask.

The proposed evaluation methodology assumes that there is only a single area in each dataset that is responsible for class selection, and we therefore created four specially prepared datasets with unambiguously designated regions of interest. The other regions/features should be assumed to be uninformative background. Our approach greatly simplifies the amount of computation required and the effort involved in tagging images when compared to ROAR or KAR. However, it requires the use of relatively simple image databases and an assurance that no hidden relation between the background features and the assigned class exists. The proposed evaluation framework can be used for fully automatic and independent evaluation of the effectiveness of the saliency maps algorithms.

### Methods under test

Thirteen techniques for saliency map generation have been compared using the evaluation method. The overview is presented in Table 1.

**Table 1:** Overview of saliency map techniques compared in our experiments.

Method	References	Abbreviation
Gradient-based visualisation	^11^	*GradientBased*
Guided back-propagation	^12^	*GuidedBackProp*
Gradient-weighted Class	^9^	*GradCAM*
Activation Mapping		
Smooth Grad	^13^	*SmoothGrad*
Guided Grad-CAM	^9^	*GuidedGradCAM*
Grad-CAM++	^15^	*GradCAM++*
Smooth Grad-CAM++	^14^	*SmoothGC++*
Fast-CAM with Grad CAM	^16^	*FastCAM-GC*
Fast-CAM with Grad CAM++	^16^	*FastCAM-GC++*
Eigen-CAM	^19^	*EigenCAM*
Eigen Grad-CAM	^19^	*EigenGradCAM*
LayerCAM	^20^	*LayerCAM*
Poly-CAM	^21^	*PolyCAM*

*Gradient-based saliency visualisation.* These saliency maps were originally described in^11^. The authors present several uses of network internal gradients such as the generation of images representative of a given class according to a class scoring model or the query of a network regarding the spatial contribution of a particular class in a given image. In the proposed approach, saliency maps are extracted after a single back-propagation pass through a DNN.

*Guided back propagation.* This method of providing a visual explanation of network decisions was proposed in^12^. It is based on the DeConvNet explanation method^7^, but it differs in the way that it handles back propagation through the rectified linear (ReLU) non-linearity^12^. Compared to the usual back propagation, guided back propagation utilizes additional signals from the higher layers of a DNN.

*Grad-CAM.* Presented in^9^, this method applies a gradient-based weighting to class activation maps. It uses gradients associated with any target concept or target class computed at the last convolutional layer to produce a coarse localization map that exposes regions of the image that are pertinent to a network’s prediction.

*Smooth Grad.* Proposed in^13^, this technique is intended to remove noise from the outcomes of Grad-CAM. It generates similar images by adding noise to the image and averages the resulting sensitivity maps to obtain a high-fidelity saliency map.

*Guided Grad-CAM* This method is a variant of Grad-CAM proposed in^9^. It combines guided back propagation^12^ with the Grad-CAM method.

*Grad-CAM++.* This variant of Grad-CAM was presented in^15^ to improve saliency maps in images containing multiple objects. The technique uses a linear combination of the positive partial derivatives of the last convolutional layer.

*Smooth Grad-CAM++.* Presented in^14^, this variant of Smooth Grad uses the Grad-CAM++ method as a baseline from which sensitivity maps are generated.

*Fast-CAM with Grad CAM.* This approach was proposed in^16^ as an efficient way to produce saliency maps. To evaluate results, the numeric measures Remove And Retrain (ROAR) and Keep And Retrain (KAR)^17^ were proposed. Using ROAR and KAR to compare saliency maps, the authors proved that Fast-CAM achieves accuracy greater than or equal to that of Grad CAM.

*Fast-CAM with Grad CAM++.* This version of Fast-CAM^16^ uses Grad CAM++ to obtain a CAM map that is then used in the computation of a saliency map.

*Eigen-CAM*: The technique, described in^19^, involves computing and visualizing the principal components of the learned features from the convolutional layers. Specifically, it focuses on computing and visualizing the first principal component of the 2D activations obtained from the network’s convolutional layers.

*Eigen Grad-CAM*: Variation of EigenCAM which incorporates class discrimination. Computes the first principal component of the activations. However, in EigenGrad-CAM, this computation is combined with the gradients.

*LayerCAM*: This method^20^ generates class activation maps for CNN-based image classifiers to improve object localization. The algorithm achieves this by assigning weights to the activations in the feature map based on the positive gradients. LayerCAM extends the capability of generating reliable class activation maps not only from the final convolutional layer, but also from shallow layers, providing more accurate localization information.

*Poly-CAM*: The method^21^ generates saliency maps by recursively merging the high-resolution activation maps from early network layers with upsampled versions of the class-specific activation maps from later layers. Additionally, the approach introduces three different methods to associate weights to each layer activation channel, measuring the impact on the network’s output when masking or unveiling the input based on the channel activation.

### Implementation details

To compare saliency map algorithms, a Python package was used. The package was based on PyTorch^22^ and provided an out-of-the-box implementation of the method under consideration. It reused the core implementations from^21,23–25^ but with slight modification for compatibility with a newer version of Python (3.8) and various network architectures such as VGG16^26^, Resnet50^27^ or SCNN^28^, which were the ones used in the experiment. The only constraint on the method is that all of the layers have to be implemented as Pytorch nn.Module^22^. To attain a final saliency map of the same shape as the input image, a bi-linear interpolation was employed.

## Experiments

### Experiment 1

**Figure 2:**
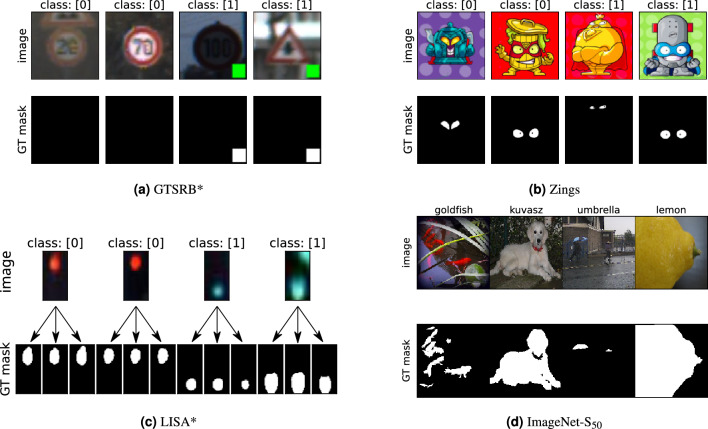
Examples of images, including generated masks for the analyzed databases.

The first experiment focused on the visual and numerical comparison of saliency maps and on checking how selected algorithms react to noise, location change, and the size change of a salient object. For the purpose of analysis, we prepared an artificial dataset based on the German Traffic Sign Recognition Benchmark (GTSRB) database^29^. This dataset, referred to as GTSRB*, contains two classes. The first class (class 0) consists of original images of traffic signs. The second class (class 1) consists of similar images with a green rectangle placed in the bottom right corner. Sample images and their corresponding GT masks from this prepared database are shown in Fig. 2a. Both the training and test GTSRB datasets were modified to contain 50% unaffected images and 50% images with the rectangle overlay. The GT masks in this experiment were easily obtained since the artifact’s exact location and size were retained and well known.

The model under consideration was always trained with the same size and location of the artifact. No additional rotations, flips or other transformations were introduced during the network training process. The goal of the experiment was to make sure that a DNN will be trained to find an object with an exact type, color and location while ignoring the other parts of the image. In this way we verify that any correctly working saliency map will indicate only the rectangular region of interest while remaining insensitive to other background features.

### Experiment 2

In experiment 2, we created a Zings dataset from images available in^30^. The dataset is composed of villains with yellow eyes and heroes with white eyes and was inspired by a popular series of children’s toys and cartoons. In contrast to our use of artificially altered images in GTSRB*, the intention for this more realistic dataset is to minimize the spurious correlations between classes. Since the drawings were designed so that the only feature distinguishing heroes from villains is eye color, we expect saliency map algorithms to clearly identify these parts of the images as the most important and that there should be no additional, hidden correlation between them. The GT masks in this experiment were obtained by manually annotating the boundaries of the eye regions in each image. Examples of the images included in this database and the respective masks that were generated are presented in Fig. 2b.

The dataset was divided into training and test datasets which respectively contained 80% and 20% of the 481 different pictures. This time data augmentation (including random rotation between − 5° and 5°, horizontal flips and vertical flips) was used to artificially expand the dataset during the training process, which helped increase the efficiency of the DNNs.

### Experiment 3

In addition to GTSRB* and Zings datasets utilized in the first two experiments, we used an additional database composed of images from the LISA Traffic Light Dataset^31,32^. This dataset contains images of real traffic lights collected under various environmental and weather conditions. From the original images we cropped only the region containing the traffic light. Only two classes from the original dataset were chosen: green (go) and red (stop). The main reason for using the traffic light dataset was that it is highly probable that there are no hidden features which might fool the classifiers since the backgrounds almost always remain black. The original division of the LISA dataset was kept, which means that two classes were taken in both training and test sets. The presented dataset is later referred to as LISA*.

Preparation of the ground truth images for the GTSRB* dataset was a trivial task. The goal was to automatically segment introduced artifact. In contrast, the segmentation of the Zings database was done fully manually, but the eyes regions were distinguishable. In case of LISA* dataset, we decided to prepare a set of masks instead of a single instance since the boundaries of light regions are blurry and various masks can be considered as correct.

The following segmentation methods were employed: (1) K-means clustering, (2) Otsu thresholding^33^ in L channel in Lab color model and in R channel in RGB color model, (3) region growing segmentation, and (4) color thresholding in HSV color space. Masks were then evaluated manually by a human operator to remove some evident failures of the segmentation algorithms. Each image was left with between one and four acceptable masks. Because it was not evident which mask should be used, we always selected the best value of the $m_{GT}$ metric from among those obtained from the possible mask realizations. Examples of images from the LISA* database and three sample masks are provided in Fig. 2c.

### Experiment 4

The purpose of the last experiment is to assess the effectiveness and quality of different saliency algorithms in capturing important visual features within images from large and real-world dataset. To accomplish this goal, we selected the ImageNet-S dataset^34^. The ImageNet-S dataset was constructed based on the well-known ImageNet dataset^35^, which contains millions of labeled images across a wide range of categories (1000 categories) and serves as a commonly used benchmark for evaluating image classification and related computer vision tasks. In order to facilitate research with limited computational resources, the authors of the study created a subset of ImageNet-S called ImageNet-S$_{50}$, consisting of 50 categories. The segmentation masks representing the ground truth were generated through a meticulous manual process. The annotation procedure involved strict labeling guidelines where annotators were divided into multiple groups, each led by a designated group leader. Following the annotation of images, the group leader consolidated all the annotations and ensured the overall quality of the annotations. Additionally, the annotations were cross-verified by other annotators within the same group, thereby ensuring a thorough quality check.

Our approach involved the utilization of three network architectures (VGG50, ResNet50 and ResNext50) that were trained using the training portion of the ImageNet dataset. In contrast to the previous experiments where SimpleCNN was used, ResNext50 was selected as a more suitable network for our experiment. We proceeded to calculate saliency maps on the validation subset of ImageNet-S$_{50}$, which consisted of 752 images across 50 categories. Subsequently, we compared the resulting saliency maps with manually segmented masks. Exemplary images and their correcponding masks are presented in Fig. 2d.

## Results

### Experiment 1

**Figure 3:**
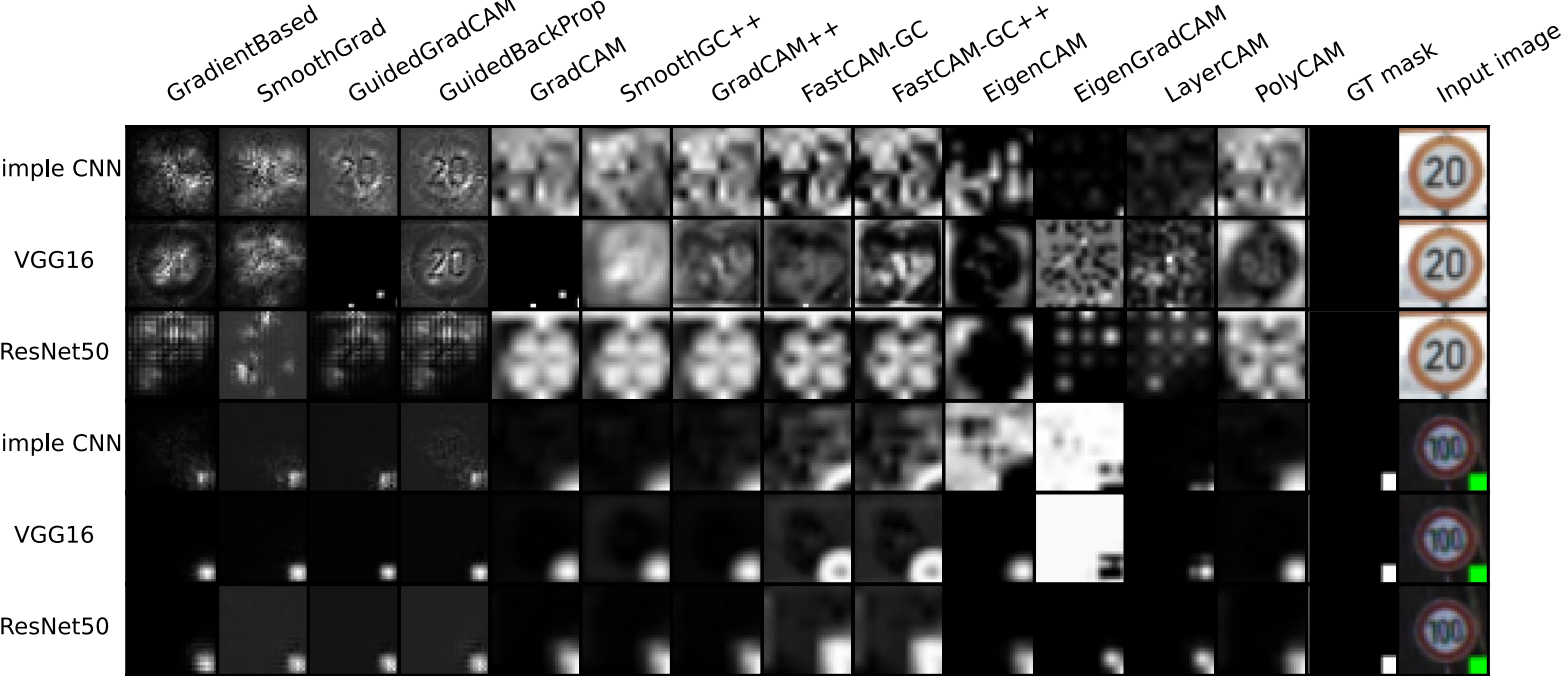
Examples of saliency maps generated for Resnet50, VGG16 and SCNN architectures, trained on GTSRB* test set. First three rows present an input image without a pattern (class 0) and the latter present an input image with a defined pattern (class 1).

**Figure 4:**
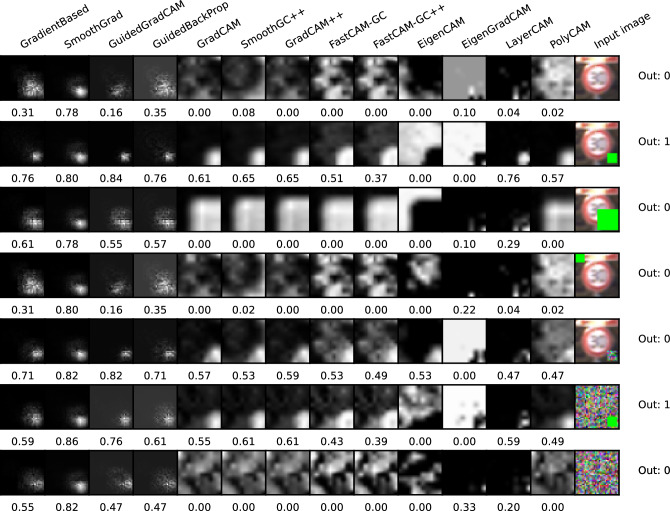
Saliency maps generated for SCNN architecture trained on GTSRB* test set. Maps are calculated for original image, image with the artifact applied and various modifications of the artifact (changing size, location and structure). The value under each picture represents the result of $m_{GT}$ measure.

Examples of saliency maps for the GTSRB* database are presented in Fig. 3. The consecutive rows show results for the three architectures used while the columns give saliency maps generated by the compared techniques. We also input GT masks and the original images in the last two columns. In Fig. 4, we present additional maps for the SCNN architecture in the case of changing artifact size and location and the application of noise to either the background or the rectangle area. While the additional maps are primarily intended for visual analysis, we have also performed calculations of the $m_{GT}$ measure for these modified examples. The results of these calculations are displayed beneath each respective saliency map in the figure. Since the whole experiment was designed to overfit the networks to the green artifact placed in the exact place and of fixed size, the $m_{GT}$ in these examples was calculated using the square mask placed as in the training set (original width and height, bottom-right corner).

**Figure 5:**
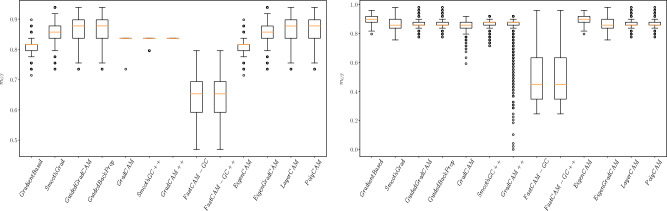
Distribution of $m_{gt}$ measure calculated for Resnet50 and VGG16 architectures, trained on GTSRB* dataset.

The mean $m_{GT}$ measures calculated for the images with squares added are presented in Table 2. The spread of $m_{GT}$ for the selected architectures (Resnet50, VGG16) can be seen in Fig. 5. Given such a simple database, the trained models achieved more than 99% accuracy on both the training and test datasets.

### Experiment 2

**Figure 6:**
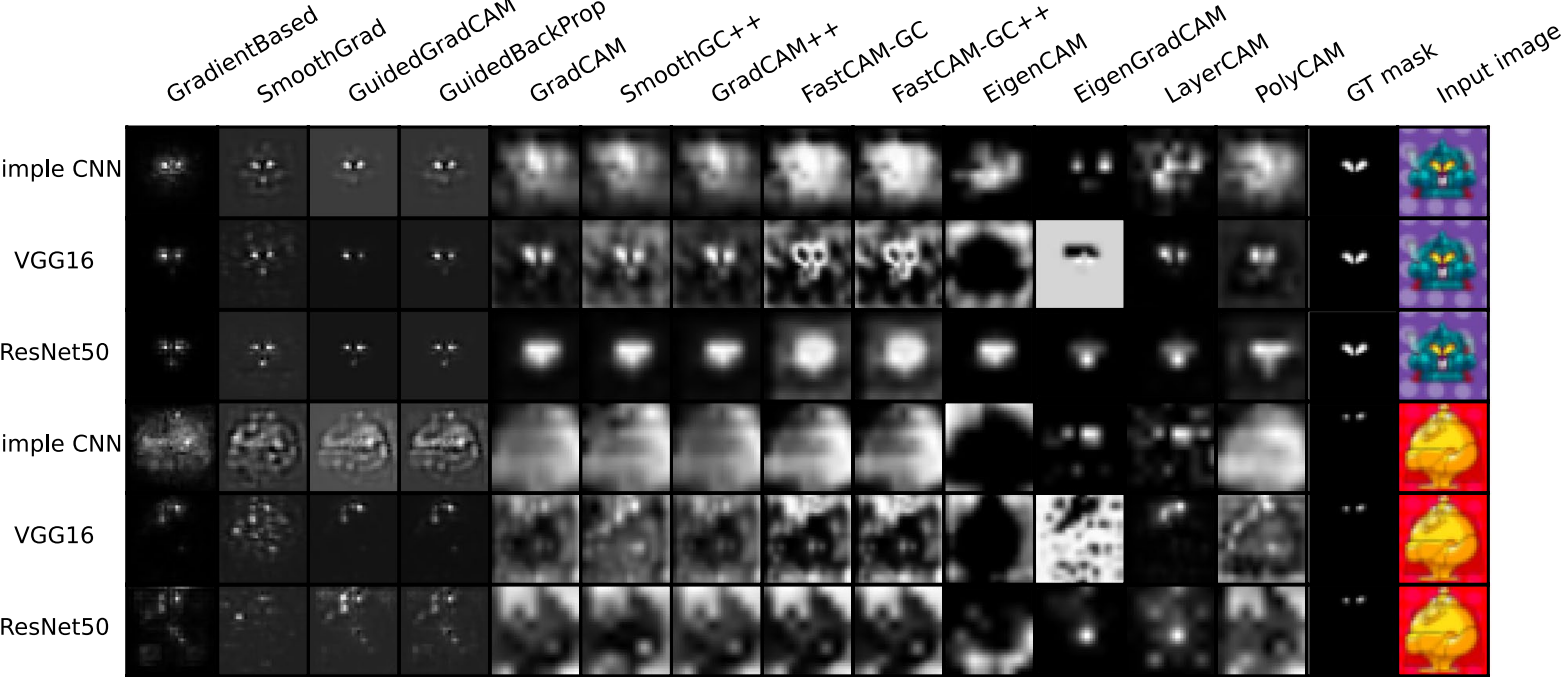
Examples of saliency maps generated for Resnet50, VGG16 and SCNN architectures, trained on Zings test set.

**Figure 7:**
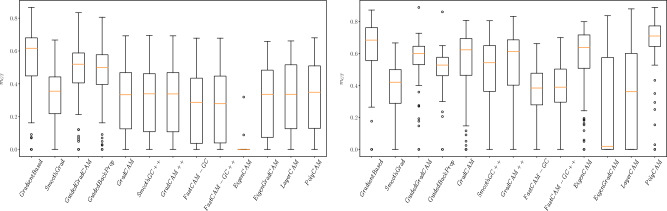
Distribution of $m_{gt}$ measure calculated for Resnet50 and VGG16 architectures, trained on Zings dataset.

Examples of saliency maps for the Zings database are presented in Fig. 6. The mean $m_{GT}$ measures from this experiment are presented in Table 2. In contrast to the data in experiment 1, the main differentiating feature is the eye region, and it is expected that the efficient saliency maps should indicate the eye region. Therefore, the $m_{GT}$ was calculated for the whole image dataset. In Fig. 7 we present the distribution of $m_{GT}$ for the selected architectures (Resnet50, VGG16) while the mean results are given in Table 2. The classification accuracy hero vs villain achieved on the Zings test dataset were $93.75\%$, $98.95\%$ and $92.70\%$ respectively for the Resnet50, VGG16 and SCNN architectures.

### Experiment 3

**Figure 8:**
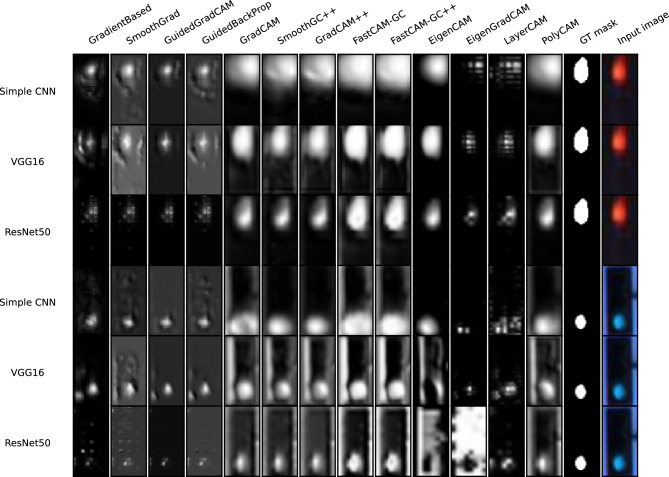
Examples of saliency maps generated for Resnet50, VGG16 and SCNN architectures, trained on LISA* test set.

**Figure 9:**
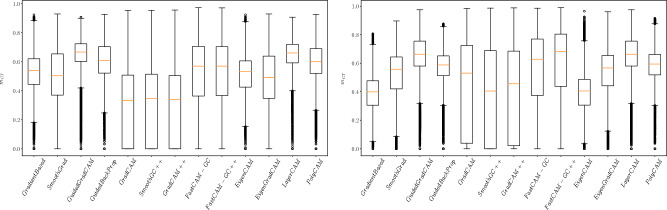
Distribution of $m_{gt}$ measure calculated for Resnet50 and VGG16 architectures, trained on LISA* dataset.

Examples of saliency maps for the LISA* database are presented in Fig. 8. The $m_{GT}$ results are given in Table 2 while the spread of results for the two selected architectures (Resnet50, VGG16) is shown in Fig. 9. As in experiment 2, all images in the dataset could be used in $m_{GT}$ calculations since the masks of both red and green lights were available. DNNs on the LISA* test dataset classifying red versus green lights achieved an accuracy of $99.96\%$, $99.98\%$ and $99.97\%$ respectively for the Resnet50, VGG16 and SCNN models. These high accuracy values indicate that the DNN models are highly effective in this particular classification task.

### Experiment 4

**Figure 10:**
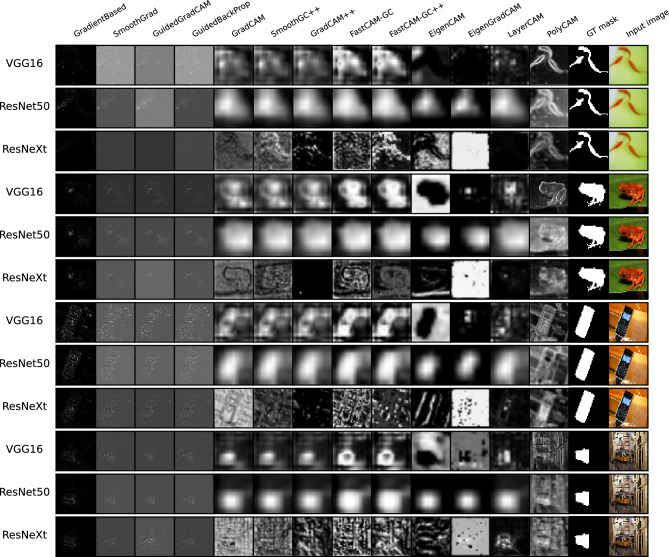
Exemplary saliency maps on ImageNet-S$_{50}$ dataset (samples of goldfish, tree frog, cellular telephone and streetcar classes).

**Figure 11:**
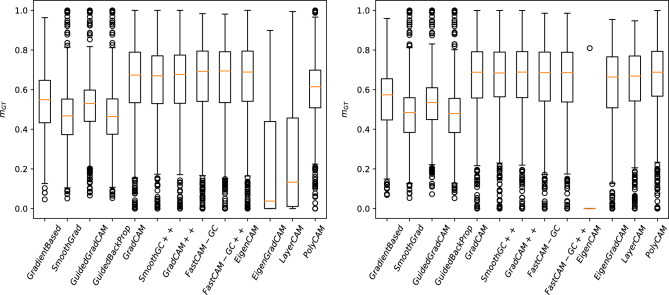
Distribution of $m_{gt}$ measure calculated for VGG16 and Resnet50 architectures, trained on ImageNet-S$_{50}$ dataset.

Examples of saliency maps for the ImageNet-S$_{50}$ database are presented in Fig. 10. As examples we chose four classes: “goldfish”, “tree frog”, “cellular telephone” and “streetcar”. The selected images represent various size of objects and background complexity. The distribution of $m_{GT}$ for two selected architectures (Resnet50, VGG16) are presented in Fig. 11. The mean $m_{GT}$ measures from this experiment are presented in Table 2. The classification accuracy of the selected models is 71.59%, 76.13% and 77.61% respectively for VGG16, Resnet50 and ResNext50 models.

## Experiment 1

In the first experiment, we wanted to ensure that the DNNs were trained to detect a specific object (the green square) at a specific and fixed location in the image. A variety of images with road signs were used as random backgrounds. Intuition suggests that the saliency maps for such trained networks should always highlight the region of the square while keeping the intensities in the remaining areas of image significantly lower. A similar effect should also be seen when analyzing images without the superimposed square object since the network should focus only on the lower right corner. Fig. 3 illustrates two exemplary results for class without a pattern (class 0) and class with a green square (class 1). As can be observed, for class zero saliency maps do not focus on any specific region and reflect traffic sign pattern. For class 1 most of the saliency maps ignore traffic sign pattern and focus on square region except Eigen Grad-Cam, which gives reverse saliency maps. This shows that for such simple and clearly defined region of interest, all saliency maps are able to focus on the expected, artifically added, pattern in the image, ignoring the edges in the background. As seen in Fig. 4 such an effect is indeed seen for most of the algorithms when applied to class 1 images, but for class 0 images, we saw the effect only for the first 4 methods, as well as LayerCAM and Eigen Grad-CAM. We did not see the lower right part of image highlighted by the remaining methods. Some random structures were seen instead. A surprising result of this experiment was that the Guided Back Propagation performed well without showing structures of images from both classes. This is contrary to findings presented in^10^.

Table 2 shows the average $m_{GT}$ score calculated for each method for images that include the green box. Two important conclusions can be drawn from the data. First, the use of maps on the much simpler SCNN results in a significant reduction in $m_{GT}$ scores. Recalling that all networks achieved similarly high levels of accuracy (above 99%), we suppose that one possible reason for the reduction is that the huge capacity of the VGG and ResNet networks allowed for more overtraining compared to the SCNN. For this reason, the network structure in these architectures was much more concentrated on a specific area in the image than it was in SCNN.

A second finding is a clear difference in the effectiveness of the methods which was particularly distinct for the more advanced networks. Looking at the results in Fig. 5, we can see that for ResNet50 and VGG16 the Fast-CAM and Fast-CAM++ methods perform significantly worse than the remaining 11 methods, which have similar $m_{GT}$ performance levels. The poor performance of these methods is also on display in Fig. 4, which shows these methods highlighting blurry regions around the region of interest while other methods were much more concentrated.

The winning methods are Eigen Grad-CAM and Guided Grad-CAM. However, the superiority is not so evident, and the differences between the $m_{GT}$ values obtained for various saliency map techniques are the smallest apart from the SCNN architecture) when compared to the outcomes of the other experiments.

Experiments involving changing the size and position of the object and including additional noise were evaluated only visually. We observed that when the green square was enlarged, the methods clearly differed in their responses. The first four methods, as well as LayerCAM and Eigen Grad-CAM, indicated the original, small area of the square, but the other 7 algorithms enlarged the area toward the size of the new object. This suggests that the methods in the second group are biased by the content of an image while those in the first group seem to maintain their reasonable results. It is worth to notice that Eigen Grad-CAM for some inputs gives reverse silency map which focuses on the pixels outside the region of interest. The superiority of Grad-based methods differs from the conclusion of^10^, which suggests that methods Gradient-based visualisation, Smooth Grad, Guided Grad-CAM and Guided back-propagation act as edge detectors. The results of our experiment, particularly when object size and position were modified, support exactly the opposite claim.

Quite similar conclusions can be drawn from experiments involving the displacement of the green square and the addition of noise in its area. As before, two groups of methods emerged with one seeming to be more effective. An interesting result was seen for the maps for images without the object present, which correspond to the first and last row in Fig. 4. The maps in the second group show completely random structures while the first four methods together with Eigen Grad-CAM and LayerCAM retained the expected indications. In the second (worse performing) group, PolyCAM deserves attention, which, although for the first image without a green square (first row) did not highlight the correct region of interest, for the second image (last row) indicated this area correctly.

## Experiments 2 and 3

In these experiments we used the more complicated Zings dataset and realistic images of traffic lights (LISA*). Analyzing the results obtained for the Zings and LISA* databases, depicted on Figs. 6 and 8, as well as in the resulting tables and plots of $m_{GT}$, we can see that the previous division of methods is not so clear and the results are more diverse.

The mentioned differences can also be seen easily in the sample maps presented in Fig. 6. The first four methods clearly indicated the eye regions while other methods indicated them only slightly (as was usually observed for GradCAM, Smooth Grad-CAM++, and Grad-CAM++) or totally missed them (as with Fast-CAM and Fast-CAM++). LayerCAM seems also to perform relatively well among the others. A similar effect can be seen in Fig. 8 for Lisa*.

The winning methods in these experiments were Grad-based, Guided Grad-CAM, Guided GradProp for Zings and Guided Grad-CAM, Eigen Grad-CAM for LISA* (similar to the effects obtained for GTSRB). The difference in average $m_{GT}$ over the analyzed set of saliency map techniques is larger in these databases than was observed in experiment 1. For the Zings database, the winning methods sometimes achieve a metric value several times larger. This observation confirms that in the case of a more realistic image databases with a less prominent sought object, performance differences are more apparent. In the case of the GTSRB* database, the green square added to an image has a very clear gradient around it, and its color stands out, so this situation may have been favorable for methods heavily biased by image content.

## Experiment 4

The experiment based on the ImageNet-S$_{50}$ collection using state-of-the-art convolutional network architectures was the closest to real-world applications of silency maps. Unfortunately, in this case, it is most difficult to assess whether the comparison methodology used is appropriate. This is due to uncertainty about any correlations of the background (or objects in the background) with the classification decision. While in previous experiments the background could be considered with a lot of confidence to be completely independent of the object, in this case such a correlation may still exist. It is also unknown if the decision on the appropriate class should be based on the entire segmented object, or perhaps on a part of it (e.g., the choice of the class “frog” may be made by the shape of the legs and not strictly on the entire object).

Nevertheless, using a similar methodology as before, we noticed that the first four methods, clearly leading in previous experiments, are noticeably worse this time. This reversal of results can be seen also for the EigenCAM and EigenGradCAM methods, which performed the worst this time as assessed by our metric. For ImageNet-S$_{50}$ dataset the best results are obtained for Grad-CAM on VGG16 architectures and LayerCAM on Res50 and ResNext50 models. Viewing the sample results in Fig. 10 it can be seen that the aforementioned two methods sometimes present the negative of the correct silency map depending on the network used. This may have been the reason for the drastic decrease in the value of the coefficient $m_{gt}$, (see the 4th and 5th rows, EigenGradCAM method, in Fig. 10).

Similarly, some of the methods that present good results included the gradient of images from the original input. It seems questionable whether such a silency map can indicate whether a given neural network model has correctly focused on an object or whether it has been overfitted.

In summary, the results of this experiment should be treated with a great deal of uncertainty, mainly due to the fact that it is not certain which feature characterizes best a given class. We cannot assume that the whole object area equally contributes to the final classifier decision. This experiment shows, in our opinion, the weakness of a benchmark base built on an image collection with complex objects and numerous classes. The previous two experiments, in our opinion, have much greater potential to be a future validation set of silency maps methods due to their simplicity and well-controlled background.

**Table 2:** Results of $m_{GT}$ measure in conducted experiments. The best result per network architecture is in bold, while the following two highest results are underlined.

Method	SCNN	VGG16	Res50
Experiment 1 (GTSRB*)			
Grad-based	0.6616	**0.8955**	0.8071
Smooth-grad	0.5760	0.8657	0.8576
Guided Grad-CAM	**0.7879**	0.8695	**0.8663**
Guided GradProp	0.6444	0.8693	0.8659
Grad-CAM	0.6550	0.8519	0.8367
Smooth Grad-CAM++	0.6626	0.8635	0.8366
Grad-CAM++	0.6558	0.8548	0.8367
Fast-CAM	0.5865	0.4893	0.6465
Fast-CAM++	0.5721	0.4906	0.6465
Eigen-CAM	0.5760	0.8657	0.8576
Eigen Grad-CAM	**0.7879**	0.8695	**0.8663**
LayerCAM	0.6444	0.8693	0.8659
Poly-CAM	0.6550	0.8519	0.8367

Method	SCNN	VGG16	Res50
Experiment 2 (Zings)			
Grad-based	**0.5332**	0.6354	**0.5330**
Smooth-grad	0.3256	0.3813	0.3273
Guided Grad-CAM	0.3544	0.5608	0.4668
Guided GradProp	0.3566	0.5023	0.4589
Grad-CAM	0.1740	0.5380	0.3088
Smooth Grad-CAM++	0.1581	0.4823	0.2986
Grad-CAM++	0.1712	0.5231	0.3080
Fast-CAM	0.2119	0.3603	0.2651
Fast-CAM++	0.2146	0.3802	0.2632
Eigen-CAM	0.0993	0.2576	0.2949
Eigen Grad-CAM	0.1707	0.3409	0.3203
LayerCAM	0.1941	**0.6647**	0.3226
Poly-CAM	0.2131	0.5769	0.3303

Method	SCNN	VGG16	Res50
Experiment 3 (LISA*)			
Grad-based	0.6052	0.3893	0.5298
Smooth-grad	0.4434	0.5388	0.4959
Guided Grad-CAM	**0.6885**	0.6279	**0.6478**
Guided GradProp	0.6427	0.5722	0.6007
Grad-CAM	0.4144	0.3803	0.3014
Smooth Grad-CAM++	0.4392	0.3260	0.3082
Grad-CAM++	0.4323	0.3382	0.3014
Fast-CAM	0.5246	0.5430	0.5315
Fast-CAM++	0.5395	0.5949	0.5307
Eigen-CAM	0.4361	0.5069	0.4751
Eigen Grad-CAM	0.6726	**0.6389**	0.6256
LayerCAM	0.6309	0.5818	0.5954
Poly-CAM	0.4155	0.4488	0.3193

Method	VGG16	Res50	ResNext50
Experiment 4 (ImageNet-S$_{50}$)			
Grad-based	0.5349	0.5493	0.4927
Smooth-grad	0.4674	0.4758	0.4294
Guided Grad-CAM	0.5162	0.5249	0.4746
Guided GradProp	0.4677	0.4744	0.4305
Grad-CAM	**0.6368**	0.6489	0.6080
Smooth Grad-CAM++	0.6273	0.6484	0.6058
Grad-CAM++	0.6312	0.6500	0.6077
Fast-CAM	0.6351	0.6403	0.6081
Fast-CAM++	0.6308	0.6411	0.6083
Eigen-CAM	0.2220	0.6111	0.5793
Eigen Grad-CAM	0.2448	0.6275	0.6042
LayerCAM	0.5871	**0.6525**	**0.6094**
Poly-CAM	0.4720	0.5508	0.5307

## Conclusions

Deep neural networks are very popular among modern researchers. One of the most common tasks of DNNs is object classification. Unfortunately, as opposed to evaluating their ability to distinguish objects in images, it is extremely difficult to determine which parts of an image affected a particular decision made by a network. A knowledge of such image regions seems crucial for identifying potential network learning errors such as a network fitting to some background elements that happen by chance to correlate with the assigned classes.

Saliency maps are supposed to be a solution to this problem. So far, several interesting techniques for generating this type of map have been presented. Unfortunately, it is still unclear to what extent the results of these algorithms indicate the real areas of interest for DNNs.

The aim of our work was to set up a series of experiments comparing the performance of saliency maps methods. The experiments varied in the classification task complexity from the detection of a simple square object superimposed on a random background (GTSRB*) through the detection of the color of traffic lights (LISA*), the analysis of eye color and the classification of objects on such a basis (as with Zings), ending on the real-world classification task on a sample 50 classes from the ImageNet database. All images in our databases were annotated fully manually or semi-automatically to unambiguously indicate the regions of the objects of interest. Further analyses applied a newly proposed evaluation procedure that compares agreement between saliency maps and object masks ($m_{GT}$). The proposed benchmarks and evaluation methodology may be further used to develop more robust saliency map algorithms.

Our study confirmed that saliency maps can be successfully used to find out what a DNN is focused on. However, different methods seem to have different performance. Some of them, mainly Fast-CAM and Fast-CAM++, showed minimal correlation between generated maps and the parts of images on which a DNN was expected to focus. Another group that includes the Grad-CAM, Grad-CAM++ and Smooth Grad-CAM++ methods showed only a partial ability to indicate the correct areas in images. However, our experiments highlighted the effectiveness of the gradient-based methods: Grad-based and Guided Grad-CAM, and they also showed that some methods may be biased by image content.

We also found that it can be tricky to prepare correct database for benchmarking the silency maps methods. The experiment on ImageNet-S$_{50}$ showed results not consistent with the comparison outcomes from the artificially prepared and much simpler images. There are a lot of unknowns that disqualifies such a complex database, with multiple classes, as a testing set.

It seems that the use of saliency maps is the way to go and that the current methods have the potential to indicate the image locations that affect a DNN’s class assignment decision. However, we would like to strongly emphasize the need to prepare benchmark experiments using carefully created image datasets. Such experiments enable an objective and quantitative comparison of methods’ effectiveness, eventually leading to the improvement in safety of neural networks classifiers and detectors.

## Data Availability

The datasets used and/or analysed during the current study available from the project leader—Krystian Radlak (krystian.radlak@pw.edu.pl)—on reasonable request.
